# Laser Impacts on Skin Rejuvenation: The Use of a Synergistic Emission of CO_2_ and 1540 nm Wavelengths

**DOI:** 10.3390/medicina59101857

**Published:** 2023-10-19

**Authors:** Stefania Belletti, Francesca Madeddu, Antonino Brando, Eugenio Provenzano, Steven Paul Nisticò, Irene Fusco, Luigi Bennardo

**Affiliations:** 1 Private Practice, Studio Kalos, 20100 Milan, Italy; sbellez@libero.it; 2 El.En. Group, 50041 Calenzano, Italy; f.madeddu@elen.it; 3 Berner Klinik Montana, 3963 Crans-Montana, Switzerland; antonino.brando@hotmail.it; 4 Department of Dermatology, Cosenza Hospital, 87100 Cosenza, Italy; e.provenzano@aocs.it; 5 Department of Dermatology, University of Rome “La Sapienza”, 00185 Roma, Italy; steven.nistico@gmail.com; 6 Department of Health Sciences, Magna Graecia University, 88100 Catanzaro, Italy

**Keywords:** laser, skin rejuvenation, wrinkles

## Abstract

*Background and Objectives*: For nearly 15 years, carbon dioxide (CO_2_) laser has been the gold standard in skin rejuvenation. Aim: The purpose of this study was to assess the efficacy of a new laser device which combines CO_2_ and 1540 nm wavelengths in wrinkles reduction, using a recently developed scanning unit named the µScan DOT scanner. *Materials and Methods*: A total of 20 female patients underwent from two to four laser treatment sessions performed once every 45–90 days. Wrinkles reduction was evaluated using the Fitzpatrick Wrinkle Classification System (FWCS). Evaluations of five-point Global Patient’s Assessment (PGA) and an Oedema and Erythema index based on a four-point scale were carried out. A careful photographic evaluation was performed to observe the patients’ aesthetic improvements. All the assessments were performed before and at 3 months follow up (3 MFU) after the last treatment session. *Results*: The mean FWCS score significantly (*p* < 0.01) decreased from 5.45 ± 2.21 at baseline to 3.3 ± 1.78 at 3 MFU after the last treatment session. In total, 8/20 (40%) subjects reported excellent improvement, 7/20 (35%) subjects showed good improvement, 5/20 (25%) subjects showed slight improvement, and 0/20 (0%) subjects showed no improvement. Concerning the Oedema index, 15/20 subjects experienced a mild one, 5/20 subjects experienced a moderate one, and none of 20 experienced a severe one, while for the Erythema index, 1/20 patients experienced mild, 18/20 patients experienced moderate, and only 1/20 patient experienced severe erythema. No severe adverse events were observed. *Conclusions*: This dual-wavelength laser technique may become a promising new option for safe, nonsurgical improvement for skin rejuvenation with an extremely low risk of scarring or hypopigmentation and shorter healing times.

## 1. Introduction

For nearly 15 years, the gold standard skin-resurfacing treatment has been the carbon dioxide (CO_2_) laser [1].

The strong dermal remodeling which results in clinical improvement after CO_2_ laser resurfacing is thought to be driven by tissue ablation and thermal coagulation at the dermal level. Scarring that may develop after CO_2_ laser treatment is caused by excessive ablation and thermal damage. However, this comes at the expense of an increased risk for scarring. Aggressive treatments with higher energies and more passes may sometimes lead to dramatic clinical results.

For these reasons, in order to avoid overly aggressive treatments, skills and expertise of the practitioner are needed. The risk of scarring has decreased compared to the original continuous wave CO_2_ laser, thanks to the development of devices using minimally ablative fractional laser therapy. In the literature, clinical observations of significant improvements in wrinkles have been observed, as well as in terms of skin laxity, acne scarring, photodamage, and facial rhytides [2,3].

The study findings of Anna Kołodziejczak et al. [4] pointed out that a series of non-ablative fractional laser therapy, bipolar radiofrequency, or intense pulse light treatment significantly affects the skin elasticity. These treatments are effective methods for the rejuvenation of periorbital skin. These treatments can be used to improve the skin elasticity and reduce wrinkles (reductions in the amount and depth of wrinkles) in the orbital and periorbital areas. The most significant improvement has been demonstrated by non-ablative fractional laser therapy. This greatest effectiveness may be related to the fact that applied laser therapy effectively affects both the elasticity and viscoelasticity of the skin. Naouri et al. [5] examined the improvement in skin tension after the application of fractional laser treatment (CO_2_ laser) in a group of 17 women.

Due to their dual ablative and thermal action, laser systems with CO_2_ sources facilitate skin remodeling by inducing the synthesis of new collagen [2,6]. Following treatment, the area where the fractional ablative laser caused immediate ablation at the epidermal or dermal level is completely replaced by invaginating epidermal cells. A microscopic ablative zone surrounds the epidermal tissue, and microscopic epidermal necrotic debris (MEND) are present in the stratum corneum. On the seventh day, MEND starts to exfoliate. One month after treatment, MEND is replaced by the normal stratum corneum [7]. These lasers are therefore efficient in treating a variety of defects and problems such as scarring and skin aging. Fractional ablative CO_2_ lasers have proven to be a promising treatment for enhancing the function and/or appearance of post-surgical scars or burn scars, as well as for skin rejuvenation [8,9,10,11]. In cultured fibroblasts, the 1540 nm wavelength is known to increase the expressions of genes related to collagen synthesis while decreasing the production of matrix proteins. [12,13,14,15].

Several published studies have already demonstrated that an extension and enhancement of the thermal effect is given by the sequential action of CO_2_ and infrared wavelengths. This assures more effective treatments in tissue remodeling, always guaranteeing the healing times needed after the fractionated emission modes, increasing cell turnover and stimulating deeper into the tissue [16,17,18,19]. In particular, recent investigations have shown that skin remodeling can be successfully treated by the simultaneous application of a CO_2_ wavelength (10,600 nm) and 1540 nm wavelength, as this synergy increases the stimulation depth and shrinkage effect [20,21].

Based on these scientific findings, the purpose of this study was to assess the efficacy of a new laser device (DuoGlide, Deka M.e.l.a Srl, Florence, Italy) which combines CO_2_ and 1540 nm wavelengths for wrinkles management, using a recently developed scanning unit named the µScan DOT scanner.

## 2. Materials and Methods

### 2.1. Patients Population

A total of 20 female patients with ages ranging between 43 and 75 years (mean age of 58.7 ± 9.2), with Fitzpatrick skin phototypes between I and III (5/20 patients presented phototype I; 11/20 patients presented phototype II; and 4/20 patients presented phototype III), were enrolled in this study.

Patients who met the inclusion criteria were those presenting medical or aesthetic indications for facial CO_2_ fractional resurfacing, wishing for a quantifiable follow-up of their laser treatment. The exclusion criteria were: subjects having recently undergone an exfoliation treatment, subjects having had surgical treatments such as lifting, and any patient with past skin disorders. Sun and UV lamp exposure were avoided prior to (at least 1 month), during, and after treatment, as were anticoagulants, retinoids, and photo-sensitizers.

### 2.2. Study Design and Laser Treatment Protocol

A dual-wavelength system (DuoGlide, DEKA M.E.L.A Srl, Florence, Italy) was used for this study. This technique relies on multi-technology combining 1540 nm and 10,600 nm CO_2_ wavelengths. Thanks to the scanner’s ability to deliver one or both wavelengths (1540 nm and 10,600 nm) in a sequential emission mode on the same DOT (the area that interests both MAZs (Microscopic Ablation Zones) and MTZs (Microscopic Thermal Zones) in the tissue), a tunable balance between ablation and coagulation depths is made possible. This allows for the provision of new and more potent treatments. One pass is made across the specific treated area using the CO_2_ + 1540 sequence. It makes the skin be simultaneously targeted by both wavelengths. For this purpose, the fractioned scanning unit (μScan DOT) was selected to perform the treatments.

The μScan DOT has a maximum scanning area of 15 × 15 mm and is an ergonomic and small scanner that allows use by even the smallest of hands.

The two wavelengths are conveyed through this scanner with different spot sizes.

The optical spot of the CO_2_ laser is about 250 µm, while that of the 1540 nm is around 1000 µm. This spot difference and the use of a spacing of 500 µm allows for non-coagulative, homogenous, and continuous heating of the entire scanning area, reaching high dermal depths. The scanner can be equipped with a contact sensor to ensure greater safety during treatment. When the μScanDOT is connected to the Duoglide, the system allows you to select different scan area shapes, different scan modes, different pulse types, dwell time, power, stack, and spacing. 

There are different scan area shapes (e.g., square and circle, etc.) to better suit the area you want to treat and different scan modes to allow, for example, the sequential emission or random emission of DOTs. The different pulse modes allow for the induction of different tissue effects. The S-Pulse mode is able to induce the homogeneous coagulation of the surrounding tissues, acting with a more circular ablation pattern, while the D-Pulse mode generates a greater shrinkage of the ablation columns and a more circumscribed thermal effect by also acting on the reticular dermis. Furthermore, due to the higher peak power of the HP, compared to the SP and DP modes described above, this mode allows for greater ablation compared to the other modes.

Finally, it is possible to change power, dwell time (the time the laser stays at a point), spacing (the distance between the various DOTs), and the stack, which serves to further increase the power.

The laser parameters for the CO_2_ wavelength source were: DP pulse, spacing between DOTs of 500–700 µm, stack 1–2, and energy/DOT 22.6–54.4 mJ, while, for the 1540 nm source, the energy/DOT was 15–20 mJ.

According to patients’ needs, the areas treated were the upper lips (4 patients), eyelids (2 patients), and the whole face (14 patients). The patients underwent 2–4 treatment sessions, and the treatments were performed once every 45–90 days. The protocol was in conformity with the ethical guidelines of the Helsinki Declaration (1975). Each patient provided informed consent regarding the dangers, advantages, and therapeutic possibilities.

### 2.3. Assessment of Efficacy

An independent dermatologist evaluated the clinical wrinkles improvement using the Fitzpatrick Wrinkle Classification System (FWCS) by comparing digital photographs taken before the treatment (baseline) and at 3 months follow up (3 MFU) after the last treatment. The Fitzpatrick Wrinkle Classification System (FWCS) assesses both wrinkles and degree of elastosis on a scale from 1 (fine wrinkles and/or mild elastosis as fine textural changes with subtly accentuated skin lines) to 9 (deep wrinkles, numerous lines with or without redundant skin folds and/or severe degree of elastosis, such as multipapular and confluent elastosis (thickened yellow and pallid) approaching or consistent with cutis rhomboidalis) [22].

In addition, the independent dermatologist was asked to determine the perceived age of the patients at 3 months follow-up after the last treatment, in order to assess the perceived rejuvenation effect.

An evaluation of a five-point Global Patient’s Assessment (PGA) with the following scores was carried out: no improvement; slight improvement; moderate improvement; good improvement; and excellent improvement. In addition, in order to monitor the skin recovery, the days needed for the expulsion of the fibrin plugs and the patient’s downtime were monitored. Finally, a careful photographic evaluation was performed to observe the patients’ aesthetic improvements. All the assessments were performed before and at the 3 months follow up (3 MFU) after the last treatment session.

### 2.4. Assessment of Safety

Oedema and Erythema indexes based on a four-point scale (none; mild; moderate; and severe) were measured at 1 week and 3 months follow up (3 MFU) after the last treatment session. All possible side effects, such as dyschromia, burning sensation, bleeding, mild to moderate posttreatment erythema, itching, crusting, and oedema, were monitored.

## 3. Results

### 3.1. Assessment of Efficacy

**Figure 1:**
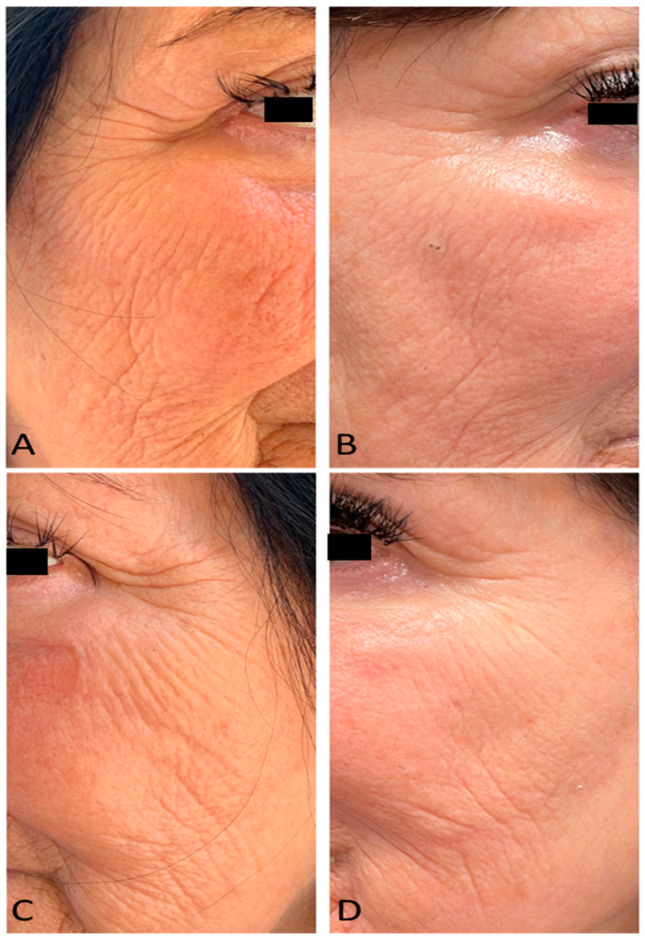
Right lateral view of a female patient’s face before (**A**) and after 4 laser treatments (3 MFU after the last treatment session) (**B**). Left lateral view of a female patient’s face before (**C**) and after 4 laser treatments (3 MFU after the last treatment session) (**D**).

All the treated patients showed significant improvements. According to the photographic assessments, a visible improvement in skin texture and a marked reduction in wrinkles in the periorbital- and cheek-treated areas were observed at the end of the treatment (3 MFU after the last treatment session) (see Figure 1).

**Figure 2:**
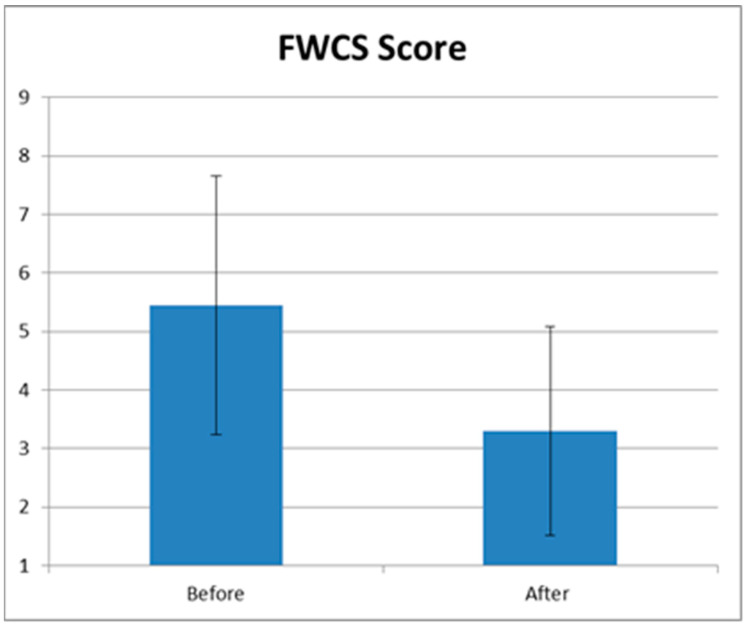
Graphical representation of mean FWCS score before and at 3 months follow up (3 MFU) after the last treatment session. A statistically significant improvement can be seen (*p* < 0.01).

These results were confirmed by the FWCS score; in fact, the mean FWCS score significantly (*p* < 0.01) decreased from 5.45 ± 2.21 at baseline to 3.3 ± 1.78 at 3 months follow up (3 MFU) after the last treatment session (Figure 2).

**Figure 3:**
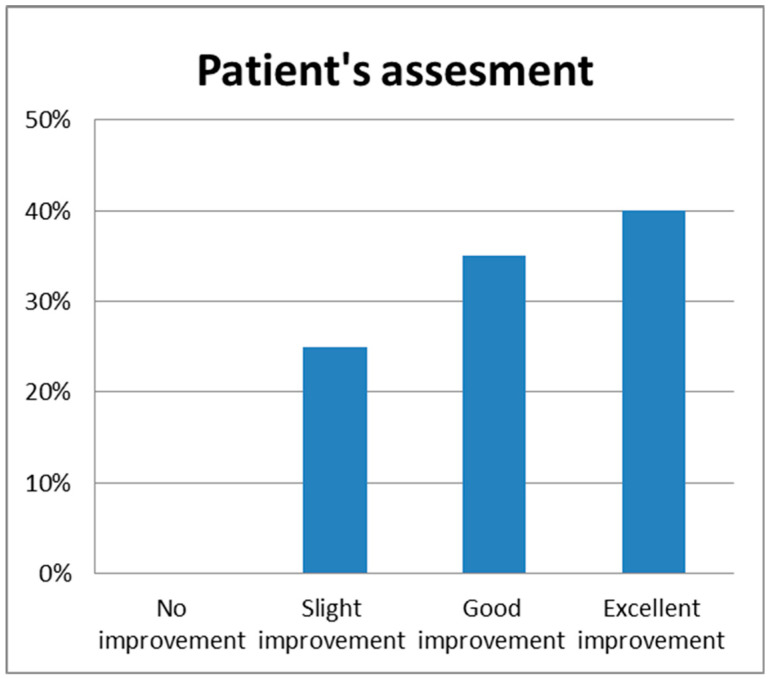
Histogram representation of Patient’s Assessment values at 3 months follow up (3 MFU) after the last treatment session.

These results were also confirmed by the patients; in fact, 8/20 (40%) subjects reported excellent improvement, 7/20 (35%) subjects showed good improvement, 5/20 (25%) subjects showed slight improvement, and 0/20 (0%) subjects showed no improvement (Figure 3).

**Figure 4:**
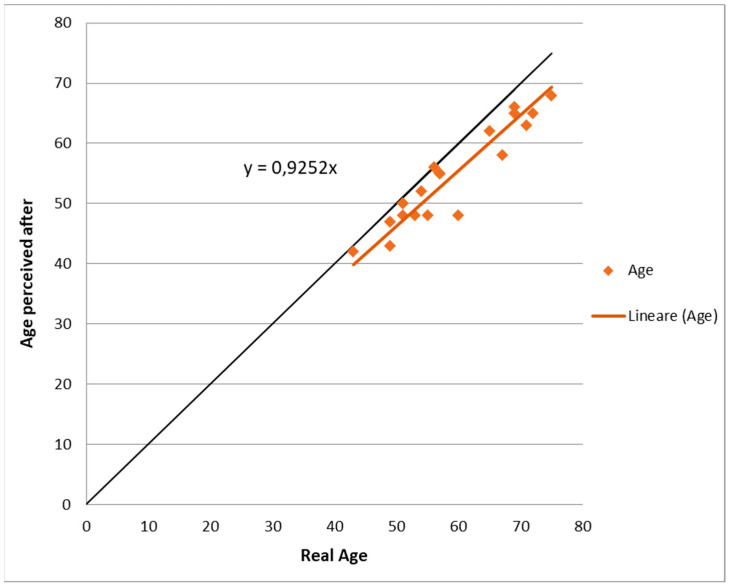
Plot of the trend’s values of perceived age of patients after laser treatment (the continuous line is the bisector, while the rhomboid dots are the perceived age of patients).

An independent dermatologist also assigned an age to each patient based on the photos taken at follow-up, and the mean difference between the patient’s perceived age at follow-up: real age was −4.3 ± 3.2 years (*p* < 0.01). In summary, based on the assessment of an independent professional, it can be said that the patients looked several years younger, and this is made even more evident by the graph in Figure 4.

If patients looked their real age, the points on the graph would be on the bisector (black continuous line), whereas, if the patients looked older after the treatment, the points would be above the bisector. What can be seen from the graph, however, is that the points are almost all below the black line, thus showing a high treatment efficacy.

Finally, the mean patient downtime was 6.2 ± 0.9 days and the average days required for the expulsion of fibrin plugs observed were 5.1 ± 0.6 days.

### 3.2. Assessment of Safety

Concerning the Oedema index, 15/20 experienced mild, 5/20 experienced moderate, and 0/20 experienced severe, while for the Erythema index, 1/20 patients experienced mild, 18/20 patients experienced moderate, and only 1/20 patient experienced severe erythema (as reported in Table 1).

The only side effects observed were slight burning in the first 24 h after laser treatment, oedema, temporary erythema, or persistent erythema and rosacea.

## 4. Discussion

The two wavelengths combined enhanced the advantages that CO_2_ laser systems already offer in terms of collagen remodeling and stimulation, as well as in terms of skin tone stimulation and strengthening. While CO_2_ works fractionally, the second wavelength of 1540 nm enabled uniform and continuous non-coagulative heating over the entire scan area, gently reaching significant depths in the dermis which could not be reached in a gentle manner by an ablative laser alone without increasing the CO_2_ energy. A recent study [23] investigated the photobiomodulation effect of 1540 nm wavelength treatment on the proliferation of cultured fibroblasts and their ability to express type I and III collagen, and the results were promising. The texture of surgical and post-traumatic scars may change as a result of non-ablative fractional lasers’ stimulation of wound healing. This means that the laser’s thermal injury is also under control, leading to a natural healing process, resulting in the growth of healthy new tissue. As a result, the skin’s appearance improves without running risks of inflammation [24,25]. The epidermis is completely protected from damage by the second wavelength, 1540 nm, which stimulates neocollagenesis. When 1540 nm is used in combination with CO_2_, its coagulative peculiarities are increased and enhanced, thus splitting the proportionality between ablation and coagulation. With the help of 1540 nm, this coagulative extension outcome under the healthy epidermis, or between the two successive CO_2_ DOTs, produces a more uniform remodeling and simulates the request that is achieved with resurfacing, but now has healing times that are comparable to fractional CO_2_ alone [20].

The findings of the current study showed a visible improvement in the patients’ skin texture and/or tightening, as well as a marked reduction in wrinkles in all treated areas without patient discomfort.

All the assessments performed in this research revealed good results, with significant improvement in FWCS scores and patients’ assessments before treatment (baseline) and at 3 months follow up (3 MFU) after the last treatment. Furthermore, by comparing the digital photographs before and after the treatment, excellent cosmetic results were achieved, with a visible aesthetic amelioration in terms of facial skin aging signs in all the examined patients. In terms of Figure 4, the difference between the trend line of the clinical data and the bisector curve would decrease as one goes down with age. However, we have no clinical data on patients younger than 43 years of age, so we will leave these evaluations to future clinical studies.

In addition, we observed that the mean number of days required for the expulsion of observed fibrin plugs was 5.1 ± 0.6 days. The use of µScan DOT with double wavelengths, in this case, decreased healing time significantly more than the traditional CO_2_ scanner (HiScan DOT scanner, DEKA M.E.L.A Srl, Florence, Italy), which, as reported in the study of Gotking et al. [19], allows for the expulsion of fibrin plugs in 14 days.

## 5. Conclusions

Based on study results, this dual-wavelength laser technique has been proven as a promising new option for safe, nonsurgical improvement for skin rejuvenation. With this innovative and correctly carried out technique, it is possible to obtain results approaching those observed with traditional CO_2_ laser resurfacing, with an extremely low risk of scarring, hypopigmentation, and shorter healing times. These laser procedures will probably ensure the possibility for patients to obtain an efficient cosmetic treatment plan thanks to their safety and a still expanding range of applications.

## Figures and Tables

**Table 1:** Oedema and Erythema Index values before and at 3 months follow up (3 MFU) after the last treatment session.

	Oedema Index	Erythema Index
None	0% (0/20)	0% (0/20)
Mild	75% (15/20)	5% (1/20)
Moderate	25% (5/20)	90% (18/20)
Severe	0% (0/20)	5% (1/20)

## Data Availability

Data that support the study findings are available on request from the corresponding author (I.F).
